# Emergence of an ST1326 (CG258) Multi-Drug Resistant *Klebsiella pneumoniae* Co-harboring *mcr-8.2*, ESBL Genes, and the Resistance-Nodulation-Division Efflux Pump Gene Cluster *tmexCD1-toprJ1* in China

**DOI:** 10.3389/fmicb.2022.800993

**Published:** 2022-03-17

**Authors:** Congcong Liu, Yuchen Wu, Yinfei Fang, Zi Sang, Ling Huang, Ning Dong, Yu Zeng, Jiayue Lu, Rong Zhang, Gongxiang Chen

**Affiliations:** ^1^Department of Clinical Laboratory, Second Affiliated Hospital of Zhejiang University, School of Medicine, Hangzhou, China; ^2^Department of Clinical Laboratory, Dali Bai Autonomous Prefecture People’s Hospital, Dali, China; ^3^Department of Clinical Laboratory Medicine, Maternal and Child Health Hospital of Linping District, Hangzhou, China; ^4^Department of Medical Microbiology, School of Biology and Basic Medical Science, Medical College of Soochow University, Suzhou, China

**Keywords:** *Klebsiella pneumoniae*, *tmexCD1-toprJ1*, ESBL genes, CG258, *mcr-8.2*

## Abstract

CG258 is the dominant carbapenemase-producing *Klebsiella pneumoniae* clone worldwide and treatment of infections caused by this clone relies largely on the last-line antibiotics, colistin, and tigecycline. However, the emergence and global dissemination of *mcr* and *tmexCD1*-*toprJ1* genes have significantly compromised their clinical applications. CG258 *K. pneumoniae* carrying both *mcr* and *tmexCD1*-*toprJ1* have not been reported. A colistin-resistant strain T698-1 belonging to ST1326, a member of CG258, was isolated from the intestinal sample of a patient and characterized by the antimicrobial susceptibility testing, conjugation assay, WGS and bioinformatics analysis. It was resistant to colistin, tetracycline, aminoglycoside, fluoroqinolone, phenicols, sulfonamide, and some β-lactams, and positive for *mcr-8.2*, *tmexCD1*-*toprJ1*, and ESBL genes (*bla*_*DHA–*1_ and *bla*_*CTX–M–*15_). The *tmexCD1*-*toprJ1* gene cluster was located in an multi-drug resistant (MDR) region flanked by Tn*As1* elements on an IncHI1B/FIB plasmid. The genetic context of *tmexCD1*-*toprJ1* was slightly distinct from previously reported Tn*5393*-like structures, with an IS*26* element disrupting the upstream Tn*5393* and its adjacent genetic elements. The *mcr-8.2* gene was inserted into the backbone of an IncFII/FIA plasmid with the genetic context of IS*Ecl1*-*mcr-8.2*-*orf*-IS*Kpn26*. To our knowledge, this is the first report of co-occurrence of *mcr-8.2* and *tmexCD1*-*toprJ1* in a CG258 *K. pneumoniae* strain. Though this strain is tigecycline sensitive, the acquisition of colistin and tigecycline resistance determinants by the endemic CG258 *K. pneumoniae* clone still poses a serious public health concern. CG258, which became resistant to multiple last resort antibiotics, would be the next emerging superbug.

## Introduction

*Klebsiella pneumoniae* is an opportunistic pathogen causing both community- and hospital acquired severe infections such as bacteremia, respiratory tract infections, and liver abscess (Gu et al., 2018; David et al., 2019). The extensive use of antibiotics has led to the emergence and rapid dissemination of multidrug resistant *K. pneumoniae*, particularly those resistant to last-line antibiotics including carbapenems, colistin, and tigecycline (Navon-Venezia et al., 2017). The majority of carbapenem-resistant *K. pneumoniae* producing KPC worldwide belong to the notorious CG258 clonal group, with ST258 and ST11 being the dominant sequence types (Dong et al., 2018). Treatment regimens for carbapenem-resistant *K. pneumoniae* are mainly reliant on colistin and tigecycline, which are classified as critically important antimicrobials by the WHO (World Health Organization) (He et al., 2019). However, the clinical potential of both antibiotics has been significantly compromised by the global dissemination of plasmid-mediated colistin-resistance genes (*mcr*) and the mobile tigecycline-resistance genes (variants of the *tet*(X), *tet*(A), *tet*(K), and *tet*(M) genes), respectively (Linkevicius et al., 2016; Liu et al., 2016; He et al., 2019; Xu et al., 2021). Recently, a novel plasmid-mediated resistance-nodulation-division (RND) efflux pump, *tmexCD1*-*toprJ1*, conferring resistance to tigecycline, quinolones, cephalosporins, and aminoglycosides was identified in *K. pneumoniae* (Lv et al., 2020). These plasmid-mediated resistance determinants are highly transmissible, presenting a severe challenge for clinical management. In this study, we reported an ST1326 multidrug resistant *K. pneumoniae* isolate, belonging to CG258, harboring resistance determinants including ESBL genes, *mcr-8.2*, and *tmexCD1*-*toprJ1* from a patient with critical illness. The emergence of colistin and tigecycline resistance determinants in the endemic *K. pneumoniae* clone constituted a true public threat.

## Materials and Methods

### Strain Origin and Antimicrobial Susceptibility Testing

An 84-year-old female patient with a previous history of pneumonia was admitted to Department of Respiratory Medicine in Dali Bai Autonomous Prefecture People’s Hospital on May 12, 2020 (Day 1) due to chronic cough. The patient experienced sudden loss of consciousness during hospitalization and received invasive mechanical ventilation *via* tracheal intubation. Anti-infective treatment with cefoperazone-sulbactam and caspofungin was also applied. She suffered from recurrent fungal and bacterial infections, fever, and gastro-intestinal bleeding. The drugs were switched to imipenem-cilastatin and ceftazidime 2 months later according to the patient’s condition. However, the patient responded poorly to the treatment and discharged on Day 96 with critical illness. On Day 104, a multidrug-resistant *K. pneumoniae* isolate (T698-1) was recovered from her intestinal samples for hospital infection surveillance. The species identity of T698-1 was confirmed by a matrix-assisted laser desorption ionization–time of flight mass spectrometer (MALDI-TOF MS) (Bruker, Germany). Antimicrobial susceptibility testing was conducted using the broth microdilution method and interpreted according to the CLSI guideline (CLSI, 2020).

### Detection of Resistance Genes and Conjugation Experiments

ESBL genes (*bla*_*TEM*_, *bla*_*CTX–M*_, *bla*_*DHA*_, *bla*_*VEB*_, *bla*_*PER*_, *bla*_*GES*_), carbapenemase genes (*bla*_*VIM*_, *bla*_*NDM*_, *bla*_*IMP*_, *bla*_*OXA*_, and *bla*_*KPC*_), plasmid-mediated colistin resistance (*mcr*) genes, tigecycline resistance determinants [*tet*(X)], and the efflux pump gene cluster *tmexCD1*-*toprJ1* were detected using previously reported primers (Dallenne et al., 2010; Poirel et al., 2011; Rebelo et al., 2018; Borowiak et al., 2020; Ji et al., 2020; Li et al., 2021). Conjugation experiments were performed by filter mating method using a rifampin resistant Escherichia *coli* EC600 (Rif*^r^*) strain as the recipient, to investigate the transferability of the *mcr* and *tmexCD1*-*toprJ1* genes. Transconjugants were selected on MacConkey agar supplemented with 600 mg/L rifampin and 1 mg/L colistin or 0.5 mg/L ciprofloxacin for transconjugants, respectively.

### Plasmid Elimination Assay

The plasmid elimination experiment was performed using sodium dodecyl sulfate (SDS, 20%) as previously described with some modifications (Liu et al., 2017). Briefly, a single colony of strain T698-1 was picked and grown in 5 ml LB broth at 37°C overnight. Then 100 μl bacterial solution was transferred to fresh LB broth containing 20% SDS and incubated at 37°C. The overnight culture was spread on LB plates, the colonies on which were streaked onto drug-free LB plates and LB plates containing 2 μg ml^–1^ colistin or 2 μg ml^–1^ tigecycline simultaneously. The colonies losing plasmids were further confirmed by PCR.

### Identification of Mutations in Colistin-Resistance Genes

Identification of mutations in *mgrB* gene was conducted using Kleborate version 2.0.4 (Lam et al., 2021). Mutations in amino acid sequences of PmrA/PmrB and PhoP/PhoQ were identified by aligning with a colistin-susceptible reference genome of the strain NJST258_1 (Genbank accession number: CP006923) reported previously (Deleo et al., 2014).

### Whole Genome Sequencing and Bioinformatics Analysis

To decipher the genomic characterization, the genome of T698-1 was extracted from overnight cultures by using the PureLink Genomic DNA Mini Kit (Invitrogen, Carlsbad, CA, United States) and sequenced using both short-read sequencing (2 × 150 bp) by Illumina Hiseq 2500 platform and long-read sequencing by Oxford Nanopore Technologies MinION platform (Li et al., 2018). Hybrid assembly of both sequencing reads was constructed using Unicycler v 0.4.4 (Wick et al., 2017). Complete genome sequence was annotated by the RAST tool and modified manually (Overbeek et al., 2013). Genotyping including species identification, multi-locus sequence typing, serotyping, identification of antimicrobial resistance genes, and virulence genes was conducted using Kleborate v2.0.4 (Lam et al., 2021). Plasmid replicons were analyzed using PlasmidFinder v2.1 (Carattoli et al., 2014). Insertion sequences (ISs) were identified using ISfinder (Siguier et al., 2006). The genetic contexts of *mcr* and *tmexCD1*-*toprJ1* genes were analyzed by comparing with similar items available in the NCBI nr (non-redundant) database. Plasmid comparisons and genetic context comparisons were visualized using BRIG and Easyfig (Alikhan et al., 2011; Sullivan et al., 2011).

## Results and Discussion

Strain T698-1 isolated from the intestinal sample of the patient was identified as *K. pneumoniae* by both MALDI-TOF MS and whole genome sequencing. It was resistant to aminoglycoside (gentamycin), fluoroqinolone (ciprofloxacin), polymyxin (colistin), tetracycline, phenicols (florfenicol, chloramphenicol), sulfonamide (trimethoprime-sulfamethoxazole), and some β-lactams (ceftazidime, cefotaxime, cefoperazone/sulbactam, ampicillin, aztreonam), but remained susceptible to carbapenems (meropenem, imipenem) and tigecycline (Table 1). In line with the resistance phenotypes, no carbapenemase gene was detected in strain T698-1, while ESBL genes including *bla*_*DHA–*1_ and *bla*_*CTX–M–*15_ were detected. Meanwhile, strain T698-1 was negative for the tigecycline resistance determinant *tet*(X), but positive for the colistin resistance gene *mcr-8.2* and the RND-type efflux pump gene cluster *tmexCD1*-*toprJ1*, which is associated with resistance to tetracyclines, quinolones, cephalosporins, and aminoglycosides. Despite carrying *tmexCD1*-*toprJ1*, strain T698-1 was susceptible to tigecycline, quite possibly due to the low expression level of this gene.

**TABLE 1:** MICs and genetic characterization of *K. pneumoniae* T698-1.

Antimicrobial agents	MIC(s)^a^ (mg/L)	Mechanism of resistance/location of resistance gene
**Aminoglycoside**		
Gentamycin	16	*aadA2*/chromosome *tmexCD1*-*toprJ1*, *strAB*, *aadA1*, *aadA2*, *aadA16*, *armA*, *aac(6′)Ib-cr*, *aph(4′)-Ia*, *aph(3′)-Ic*, *aac(3′)-IVa*/plasmid
**β-lactam**		
Meropenem	≤1	–
Imipenem	≤1	–
Ampicillin	>64	Intrinsic resistance (*bla*_*SHV–*11_/chromosome)
Aztreonam	>32	*bla*_*DHA–*1_, *bla*_*CTX–M–*15_, *bla*_*TEM–*1*B*_/plasmid
Cefotaxime	>16	*bla*_*DHA–*1_, *bla*_*CTX–M–*15_, *bla*_*TEM–*1*B*_/plasmid
Ceftazidime	>16	*bla*_*DHA–*1_, *bla*_*CTX–M–*15_, *bla*_*TEM–*1*B*_/plasmid
Cefoperazone/sulbactam	64/32	*bla*_*DHA–*1_, *bla*_*CTX–M–*15_, *bla*_*TEM–*1*B*_/plasmid
**Fluoroqinolone**		
Ciprofloxacin	>4	*tmexCD1*-*toprJ1*, *qnrB4*/plasmid ParC (80I), GyrA (83I)/chromosome
**Polymyxin**		
Colistin	64	*mcr-8.2*/plasmid
**Tetracycline**		
Tetracycline	>32	*tmexCD1*-*toprJ1*, *tet*(A)/plasmid
**Glycylcycline**		
Tigecycline	≤0.5	–
**Phenicols**		
Florfenicol	>16	*floR*/plasmid
Chloramphenicol	>64	*cmlA1*, *floR*, *catA2*/plasmid
**Sulfonamide**		
Trimethoprim-sulfamethoxazole	>16/304	*sul1*/chromosome *sul1*, *sul2*, *sul3*/plasmid
**MLS–Macrolide, Lincosamide and Streptogramin B**		
Not included in the AST panel	Na	*mph*(E) and *msr*(E)/plasmid

*^a^\Na, not applicable.*

The genome of strain T698-1 (BioProject number: PRJNA747739) was assembled into four complete circularized contigs, including a 5,265,236 bp chromosome (CP079781) encoding 5207 predicted ORFs with a GC content of 57.6% and three multi-drug resistant (MDR) plasmids (pKPT698-tmexCD, pKPT698-mcr, pKPT698-tetA). Strain T698-1 belonged to ST1326 (*gapA*-*infB*-*mdh*-*pgi*-*phoE*-*rpoB*-*tonB* allele number 3-3-1-1-1-1-16), which is closely related to the endemic *K. pneumoniae* clones ST258 (3-3-1-1-1-1-79) and ST11 (3-3-1-1-1-1-4) in clonal group (CG) 258 with only one allele (*tonB*) variance among the three STs. According to the previous study, CGs were defined as groups for which MLST profiles showed only one allelic mismatch with at least one other member of the group, suggesting strain T698-1 belonged to the notorious CG258 clonal group (Bialek-Davenet et al., 2014). To date, 77 immunologically distinct *Klebsiella* capsule types (K1-K77) have been defined by serology, and comparative analysis of the full-length capsule synthesis loci (K-loci) extracted from whole genome sequences identified 134 distinct K-loci (KL1-KL134) (Wyres et al., 2016). Strain T698-1 belonged to KL102, which has not been verified with traditional immunoelectrophoresis techniques. Apart from the *bla*_*SHV–*11_ gene conferring intrinsic ampicillin resistance in *K. pneumoniae*, the chromosome of strain T698-1 carried acquired antimicrobial resistance genes *sul1* and *aadA2*, which were bordered by mobile elements (IS*26*-IS*6100*-*orf1*-*sul1*-*orf2*-*aadA2*-IS*26*). Fluoroquinolone resistance-associated mutations including S80I in ParC and S83I in GyrA were detected in the chromosome of strain T698-1 (Wyres et al., 2019).

**FIGURE 1:**
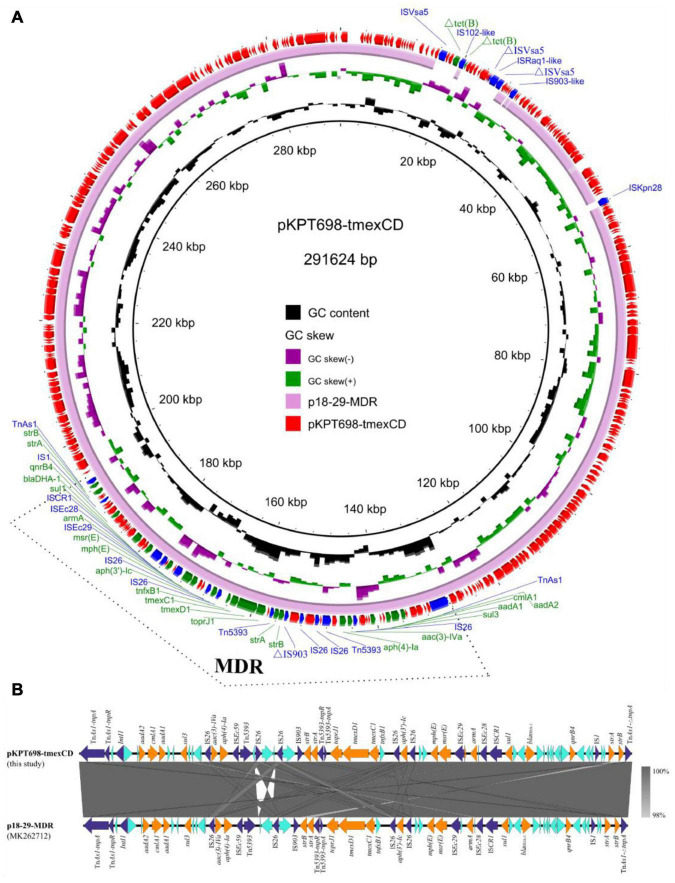
**(A)** Circular plasmid map of pKPT698-tmexCD. Green, blue, and red arrows indicate the antimicrobial resistance genes, mobile elements, and other predicted ORFs, respectively. The names of antimicrobial resistance genes, mobile elements are labeled alongside the corresponding arrows. The multi-drug resistant (MDR) region is indicated with dotted line frame. **(B)** Linear comparisons of the MDR region in plasmids pKPT698-tmexCD and p18-29-MDR. Cyan, blue, and yellow arrows indicated the antimicrobial resistance genes, mobile elements, and other predicted ORFs, respectively.

Plasmid pKPT698-tmexCD (CP079784) was 291,624 bp in length, with an average G + C content of 46.8%. It is an IncHI1B/FIB plasmid comprising 330 predicted ORFs. One plasmid, p18-29-MDR (MK262712), with similar backbone of 99.94% identity at 99% coverage to pKPT698-tmexCD was retrieved in the NCBI nr database by BLASTn analysis (Figure 1A). Plasmids pKPT698-tmexCD and p18-29-MDR, both from *K. pneumoniae*, shared the same backbone and plasmid replication genes, indicating that they might be originated from a common ancestor and have undergone evolution separately. A total of 18 different resistance genes containing *tmexCD1*-*toprJ1*, *aadA1*, *aadA2*, *cmlA1*, *sul3*, *aac(3)-IVa*, *aph(4)-Ia*, *aph(3′)-Ic*, *strA* (two copies), *strB* (two copies), *mph*(E), *msr*(E), *armA*, *sul1*, *bla*_*DHA–*1_ and *qnrB4*, and an truncated IS*102*-like gene, △*tet*(B), were identified on plasmid pKPT698-tmexCD. Antimicrobial resistance genes were located on a 65,340 bp MDR-encoding region bordered by the Tn*As1* transposon. A further BLAST search of this region in the NCBI nr database returned seven plasmid hits (CP076031, MK262712, MN099026, MT647838, AP023338, CP075257, CP075287) with >99.7% identity and ≥95% coverage, indicating this MDR region was most likely acquired by horizontal gene transfer, and it was transmissible among diverse plasmid backbones. The MDR region harbored an array of mobile elements including Tn*As1*, Tn*5393*, IS*Ec28*, IS*Ec29*, IS*Ec59*, IS*903*, IS*CR1*, IS*1*, 5 copies of IS*26*, and an *IntI1* integrase, suggesting active genetic recombination could occur in this region (Figure 1B). The RND-type efflux pump gene cluster, *tmexCD1*-*toprJ1*, was first reported to be located on the transposon Tn*5393* of plasmid pHNAH8I-1 (MK347425), which was considered to be associated with its translocation (Lv et al., 2020). On plasmid pKPT698-tmexCD, the upstream Tn*5393* and its adjacent genetic elements were disrupted by an IS*26*, constituting the genetic context of IS*26*-*tnfxB1*-*tmexC1*-*tmexD1*-*toprJ1*-Tn*5393*:*tnpA*-Tn*5393*:*tnpR*-*strA*-*strB*, which was slightly distinct from that reported previously (Lv et al., 2020; Sun et al., 2020). Predicted to have originated from the chromosome of *Pseudomonas* sp., the RND efflux pump gene cluster *tmexCD1*-*toprJ1*, as well as its variants *tmexCD2*-*toprJ2* and *tmexCD3*-*toprJ3*, has been reported from a wide diversity of species including *Pseudomonas* sp., *K. pneumoniae*, *Klebsiella quasipneumoniae*, *Raoultella ornithinolytica*, and *Proteus mirabilis* (Lv et al., 2020; Sun et al., 2020; Wan et al., 2020; Li et al., 2021; Wang C.-Z. et al., 2021; Wang Q. et al., 2021). Diverse mobile genetic elements including plasmids, insertion sequences, transposons, integrative, and conjugative elements (ICEs) were reported to have contributed to its wide dissemination (Lv et al., 2020; Wan et al., 2020; Hirabayashi et al., 2021; Wang Q. et al., 2021). Plasmid elimination was performed to demonstrate the influence of TMexCD1-TOprJ1 and MCR-9 to tigecycline and colistin resistance, respectively. Despite trying three times, we failed to get a single plasmid-eliminated derivative strain and only the strain T698-1-PC of which both pKPT698-tmexCD and pKPT698-mcr were cured of was obtained. The T698-1-PC showed the same or close MIC value to tigecycline (≤0.5 mg/L), tetracycline (32 mg/L), quinolones (>4 mg/L), gentamycin (>16 mg/L), and cephalosporins (>16 mg/L) as the original strain T698-1 and those antibacterial agents were the substrate spectrums of TMexCD1-TOprJ1 for *K. pneumoniae* (Lv et al., 2020), further proving the inefficacy of TMexCD1-TOprJ1 in T698-1.

**FIGURE 2:**
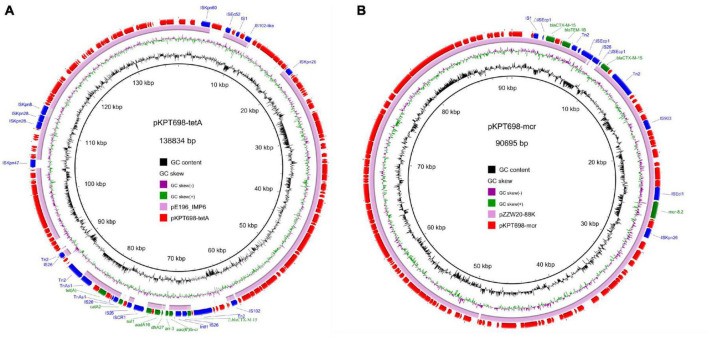
Circular plasmid maps of pKPT698-tetA **(A)** and pKPT698-mcr **(B)**. Green, blue, and red arrows indicate the antimicrobial resistance genes, mobile elements, and other predicted ORFs, respectively. The names of antimicrobial resistance genes, mobile elements are labeled alongside the corresponding arrows. **(A)** The MDR region of pKPT698-tetA including *aac(6′)Ib-cr*, *arr-3*, *dfrA27*, *aadA16*, *sul1*, and *catA2* was bordered by the transposable element Tn*2*. **(B)** pKPT698-mcr carried *bla*_*CTX–M–15*_ (two copies), *bla*_*TEM–1B*_, *mcr-8.2* with mobile elements such as IS*903B* and IS*Ecl1* inserting into this segment.

The second plasmid, pKPT698-tetA (CP079782), is a 138,834 bp, IncFIB plasmid which encodes 167 predicted ORFs with a G + C content of 52.3%. BLASTn analysis in the NCBI nr database returned no hits with similar backbones (>85% coverage), and the most closely related plasmid (pE196_IMP6, 250,392 bp, AP019405) exhibited 99.49% identity to pKPT698-tetA at 80% coverage. pKPT698-tetA harbored multiple antimicrobial resistance genes including *aac(6′)Ib-cr*, *arr-3*, *dfrA27*, *aadA16*, *sul1*, and *catA2* located in a 23,779 bp MDR region bordered by the transposable element Tn*2*. The MDR region is >99.95% identical to its counterparts on plasmid pKp21774-135 (MG878868) from *K. pneumoniae* and plasmid pM206-NDM1 (AP018830) from *Enterobacter xiangfangensis* at 100% coverages, suggesting this region was acquired by horizontal gene transfer (Figure 2A).

The third plasmid, pKPT698-mcr (CP079783), is a 90,695 bp, IncFII/FIA plasmid which encodes predicted 112 ORFs with a G + C content of 50.1%. It was 99.97% identical to plasmid pZZW20-88K (accession: CP058962) at 98% coverage. Antimicrobial resistance genes carried by pKPT698-mcr include *bla*_*CTX–M–*15_ (two copies), *bla*_*TEM–*1*B*_, and *mcr-8.2* (Figure 2B). Several mobile elements such as IS*903B* and IS*Ecl1* were reported to have played pivotal roles in the dissemination of *mcr-8* among Enterobacteriaceae (Wang et al., 2018; Zhang et al., 2021). In line with the previous findings, the *mcr-8.2* gene on pKPT698-mcr was associated with the genetic context of IS*Ecl1*-*mcr-8.2*-*orf*-IS*Kpn26* (Zhang et al., 2021). Both pKPT698-tmexCD and pKPT698-mcr were shown to be non-conjugative under laboratory conditions. The elimination of pKPT698-mcr in strain T698-1-PC caused a marked decrease of MIC values to colistin from 64 mg/L in T698-1-PC to 1 mg/L in T698-1, indicating *mcr-8.2* genes mediated colistin resistance in T698-1. Besides, we further screened common chromosomal mutations that caused colistin resistance. The result from Kleborate showed that no mutations associated with resistance to colistin were detected in *mgrB* gene. Compared with the colistin-susceptible genome of the strain NJST258_1, T698-1 did not possess any amino acid mutations of PmrA/PmrB, and PhoP/PhoQ. Therefore, these data suggested that the *mcr-8.2*-bearing plasmid in this strain is responsible for colistin resistance.

The plasmid-borne tigecycline-resistant RND efflux pump gene cluster *tmexCD1-toprJ1* was first reported in *K. pneumoniae* in China in 2020 (Lv et al., 2020), with an extremely high prevalence of 52.4% in animals and 0.08∼2.5% in patients (Lv et al., 2020; Sun et al., 2020). This indicated that we should be alert to the further dissemination of TmexCD1-ToprJ1-positive *K. pneumoniae* in hospital despite that only one *K. pneumoniae* carrying TmexCD1-ToprJ1 was detected. Worse still, the *K. pneumoniae* strains even plasmids co-harboring *tmexCD1-toprJ1* and *mcr* have been prevailing in China, and some strains belonged to the ST11 clone, the predominant and high risk clone of CRKP strains in China (Sun et al., 2020). The simultaneous presence of NDM carbapenemases further promoted the emergence of pan-drug resistant bacteria, especially in *K. pneumoniae* with high genome plasticity (Sun et al., 2020). The MDR strain T698-1 was susceptible to tigecycline and carbapenems but conferred resistance to colistin on account of *mcr-8.2*, a variant of the *mcr-8* colistin resistance gene identified in 2020 (Ma et al., 2020), suggesting that future nosocomial infections surveillance pay more attention to *K. pneumoniae*.

## Conclusion

In conclusion, we reported the emergence of a ST1326 MDR *K. pneumoniae* strain in a hospital in China, which belonged to the endemic CG258 clone -and carried *mcr-8.2*, ESBL genes, and the RND efflux pump gene cluster *tmexCD1*-*toprJ1*. *K. pneumoniae* of CG258 was commonly shown to be associated with carbapenem resistance. The acquisition of colistin and tigecycline resistance determinants by CG258 *K. pneumoniae* may pose a serious public health concern.

## Data Availability Statement

The complete genome sequence of strain T698-1 was deposited in the GenBank database under the BioProject number: PRJNA747739, with accession numbers CP079781-CP079784.

## Ethics Statement

The study was approved by the Ethics Committee of Second Affiliated Hospital, Zhejiang University School of Medicine (2020-319). Written informed consent was obtained from the patients for the publication of any potentially identifiable data.

## Author Contributions

CL and YW performed the experiments and wrote the original draft. YF and ZS contributed to data analysis and manuscript writing. LH, ND, YZ, and JL helped with the experiments. RZ helped editing the manuscript and contributed to study design. GC put forward the conception, designed the study, and edited the manuscript. All authors contributed to the article and approved the submitted version.

## Conflict of Interest

The authors declare that the research was conducted in the absence of any commercial or financial relationships that could be construed as a potential conflict of interest.

## Publisher’s Note

All claims expressed in this article are solely those of the authors and do not necessarily represent those of their affiliated organizations, or those of the publisher, the editors and the reviewers. Any product that may be evaluated in this article, or claim that may be made by its manufacturer, is not guaranteed or endorsed by the publisher.
